# Integrative analysis of circadian clock with prognostic and immunological biomarker identification in ovarian cancer

**DOI:** 10.3389/fmolb.2023.1208132

**Published:** 2023-06-20

**Authors:** Lianfang Zhao, Yuqin Tang, Jiayan Yang, Fang Lin, Xiaofang Liu, Yongqiang Zhang, Jianhui Chen

**Affiliations:** ^1^ Prenatal Diagnosis Center, Suining Central Hospital, Suining, Sichuan, China; ^2^ Clinical Bioinformatics Experimental Center, Henan Provincial People’s Hospital, Zhengzhou University, Zhengzhou, China; ^3^ Guangzhou Women and Children’s Medical Center, Guangzhou Medical University, Guangzhou, China

**Keywords:** circadian clock, immune microenvironment, prognosis, biomarker, ovarian cancer

## Abstract

**Objective:** To identify circadian clock (CC)-related key genes with clinical significance, providing potential biomarkers and novel insights into the CC of ovarian cancer (OC).

**Methods:** Based on the RNA-seq profiles of OC patients in The Cancer Genome Atlas (TCGA), we explored the dysregulation and prognostic power of 12 reported CC-related genes (CCGs), which were used to generate a circadian clock index (CCI). Weighted gene co-expression network analysis (WGCNA) and protein-protein interaction (PPI) network were used to identify potential hub genes. Downstream analyses including differential and survival validations were comprehensively investigated.

**Results:** Most CCGs are abnormally expressed and significantly associated with the overall survival (OS) of OC. OC patients with a high CCI had lower OS rates. While CCI was positively related to core CCGs such as *ARNTL*, it also showed significant associations with immune biomarkers including CD8^+^ T cell infiltration, the expression of *PDL1* and *CTLA4*, and the expression of interleukins (*IL-16*, *NLRP3*, *IL-1β,* and *IL-33*) and steroid hormones-related genes. WGCNA screened the green gene module to be mostly correlated with CCI and CCI group, which was utilized to construct a PPI network to pick out 15 hub genes (*RNF169*, *EDC4*, *CHCHD1*, *MRPL51*, *UQCC2*, *USP34*, *POM121*, *RPL37*, *SNRPC*, *LAMTOR5*, *MRPL52*, *LAMTOR4*, *NDUFB1*, NDUFC1, *POLR3K*) related to CC. Most of them can exert prognostic values for OS of OC, and all of them were significantly associated with immune cell infiltration. Additionally, upstream regulators including transcription factors and miRNAs of key genes were predicted.

**Conclusion:** Collectively, 15 crucial CC genes showing indicative values for prognosis and immune microenvironment of OC were comprehensively identified. These findings provided insight into the further exploration of the molecular mechanisms of OC.

## 1 Introduction

With 207,252 deaths worldwide in 2020 (Sung et al., 2021), the mortality rate of ovarian cancer is ranked first among gynecological malignant tumors. The characteristics of insidious symptoms, high degree of malignancy, and easy metastasis of ovarian cancer make it a great challenge for early screening. Nearly 70% of OC patients are diagnosed at an advanced stage (Cho and Shih Ie, 2009). Moreover, the pathogenesis and metastasis mechanism of OC remain elusive, so it is particularly urgent to identify new biomarkers and potential drug targets for improving the clinical outcomes of OC patients.

Circadian rhythm is a physiological cycle phenomenon of about 24 h in mammalian body. Accumulating evidence has elucidated the significance of circadian rhythm composed of central and peripheral clocks in harmony with the external environment (LeGates et al., 2014; Jagannath et al., 2017). *BMAL1* and *CLOCK* form a dimer, which promotes the expression of cryptochrome (*CRY1* and *CRY2*) and period (*PER1*, *PER2*, and *PER3*) genes. *CRY* and *PER* form a complex that enters the nucleus and suppresses the *CLOCK*–*BMAL1* complex (Etchegaray et al., 2003; Hirayama et al., 2007; Nader et al., 2009). This forms the transcription-translation loop of the central clock. Nuclear receptors *REV*-*ERBα* and retinoic acid-like orphan receptor α (*RORα*) regulate the ROR element on the *BMAL1* promoter, providing a stable oscillation cycle (Badiu, 2003). The circadian clock gene is the molecular basis for the circadian clock to produce and maintain circadian rhythms. At present, increasing evidence has found that more than ten circadian clock genes form transcriptional translation loops, including *ARNTL*, *CLOCK*, *CRY1*, *CRY2*, *NR1D1*, *NR1D2*, *PER1*, *PER2*, *PER3*, *RORA*, *RORB*, *RORC*, etc. (Yang et al., 2017). Target genes regulated by circadian clock genes are called clock control genes, which are effector molecules of the circadian clock. In mammals, clock control genes play an important role in maintaining physiological homeostasis (e.g., hormone secretion, cell growth, proliferation, and cell metabolism) (Udo et al., 2004).

A growing number of studies strongly support the existence of crosstalk between cancer and the circadian clock (Battaglin et al., 2021). Increasing data have suggested that circadian clocks exerted a vital function in the regulation of cancer-related physiological systems, such as cell proliferation, cell apoptosis, DNA injury and repair, and metabolism (Peyric et al., 2013; Dakup and Gaddameedhi, 2017). The imbalance of the circadian clock particularly affects the progression of endocrine-related cancers including cervical, prostate, and ovarian cancers by dysregulating key hormone levels (Morales-Santana et al., 2019; Hadadi and Acloque, 2021). Aberrant expression of *ARNTL* and *CRY1* was found in OC cell lines (Tokunaga et al., 2008). Moreover, the low expression of *CRY1* and *BMAL1* was reported to be associated with the poor survival of OC patients (Tokunaga et al., 2008), and the overexpression of *BMAL1* could inhibit the cell growth of OC (Yeh et al., 2014). But controversy still exists. For existence, a recent study did not find the increased risk of ovarian cancer for night-shift work (Dun et al., 2020). Thus, a comprehensive understanding of circadian clock is required to better evaluate its critical role in the carcinogenesis of OC, which will enable the identification of clinical biomarkers and molecular targets.

**FIGURE 1:**
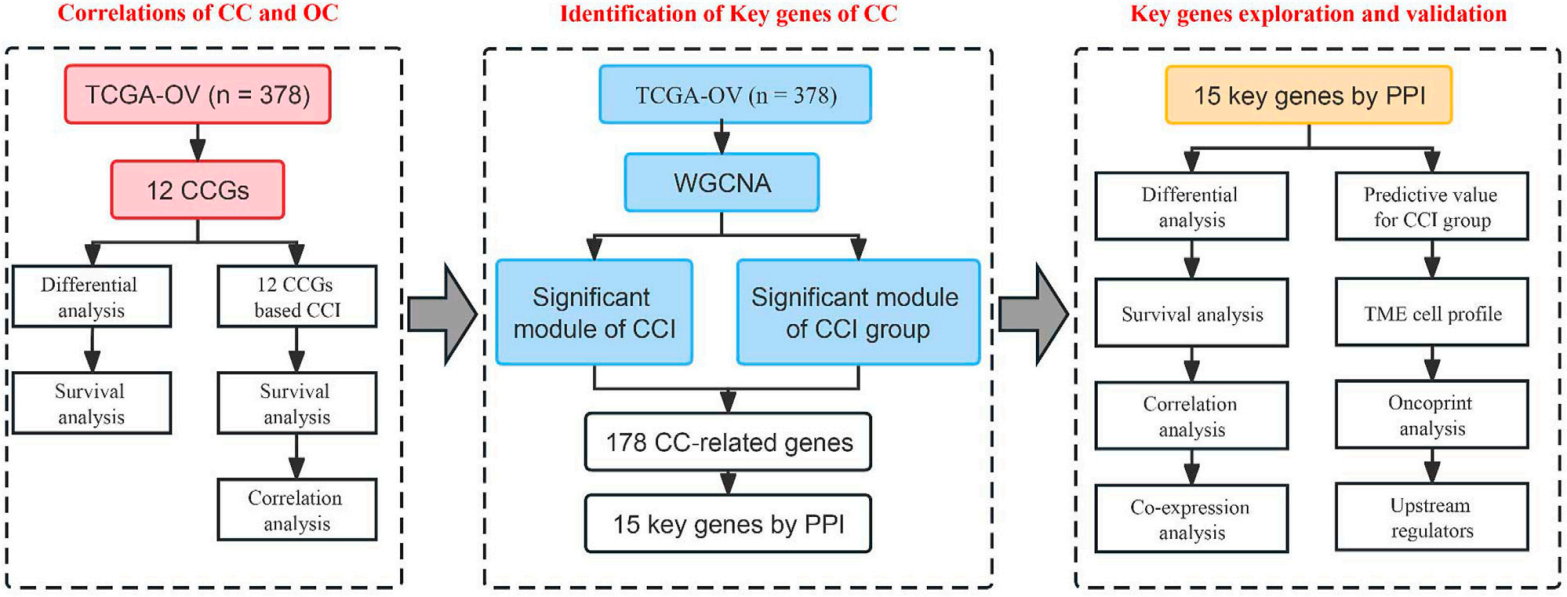
Workflow of this study.

Moreover, previous studies indicated that circadian clock gene disruption contributes to independent risk factors for tumor microenvironment (TME) (Aiello et al., 2020; Xuan et al., 2021). However, the specific functions of circadian clock in the prognosis and therapy of OC are still unclear. Therefore, this study aimed to elucidate the vital role of the circadian clock in OC using multi-omics methods. We integratively identified 15 potential key genes related to CC, which showed great correlations to the infiltration levels of multiple tumor immune microenvironment (TIME) cell types. These data strengthened the linkage of CC and tumor immune status of OC, and extended the understanding of its molecular mechanism, and survival analysis results suggested their good potential in the future development of new prognostic biomarkers and immunotherapy targets. The workflow of this study is shown in Figure 1.

## 2 Materials and methods

### 2.1 Data collection and preprocessing

We downloaded the gene-expression dataset (TCGA-OV) and the complete OS data of 378 OC in the TCGA database from the UCSC XENA project (https://xena.ucsc.edu). The disease-specific survival (DSS) and corresponding clinicopathologic characteristics including age, stage, grade, tumor status, and lymphatic invasion status were also achieved. The RNA-seq data were preprocessed as we previously reported (Zhang et al., 2022). 12 literature-derived CCGs in basic mammalian central feedback loop were collected (*ARNTL*, *CLOCK*, *PER1*, *PER2*, *PER3*, *CRY1*, *CRY2*, *NR1D1*, *NR1D2*, *RORA*, *RORB*, and *RORC*) (Shen et al., 2020; Cash et al., 2021). Gene expression levels between the tumor and adjacent normal tissues were compared using the online GEPIA database (Tang et al., 2017).

### 2.2 Correlations of CCI with the prognosis and TIME of OC

Based on the TCGA-OV dataset, we generated a circadian clock index to comprehensively represent the overall activity of the CC status, which was computed as the average RNA-seq z-scores of the 12 core CCGs for each sample. All patients were designated into the high- and low-CCI groups depending on the optimal cutoff for the Kaplan-Meire survival analysis. The single sample gene set enrichment analysis (ssGSEA) algorithm was applied to infer the activated CD8 T cell infiltration by the “GSVA” package (Bindea et al., 2013; Charoentong et al., 2017; Zhang et al., 2022; Guo et al., 2023). Scatter plots were drawn to determine the correlations of CCI with TIME signatures including activated CD8 T cell infiltration, the expression of well-known immunotherapeutic targets such as *PDL1* and *CTLA4*, and steroid hormones-related genes. The correlations between CCI and multiple interleukins including the *IL-1* superfamily, *IL-6* family, *IL-10* family, *IL-17* family, and other interleukins were also evaluated.

### 2.3 WGCNA and key module identification

We construct a co-expression network with the “WGCNA” package in R (Langfelder and Horvath, 2008; Tang et al., 2022b). First, we filtered the median absolute deviation (MAD) top 5,000 genes for sample clustering (Tang et al., 2022b). Next, we select the best soft-thresholding power to ensure the scale-free topology (*R* ^2^ > 0.9). The adjacency was then transformed into a topological overlap matrix (TOM) by using TOM similarity and the corresponding dissimilarity (dissTOM). Finally, at least 30 co-expressed genes were aggregated into different MEs by the dynamic tree-cut method. We decided on a cut line (0.3) for merging highly similar modules to make the modules more compact (Zhang et al., 2022). To determine their relevance to clinical traits, Pearson correlations between module eigengenes and clinical phenotypes were calculated and were shown with a correlation heatmap. In this study, we chose the most significant module that correlated with CCI and CCI group for further analysis, and gene significance (GS) and module membership (MM) were also calculated. The CC-related genes were determined as those with MM > 0.5 and GS > 0.4 in the most significant module.

### 2.4 PPI network construction and key genes identification

The PPI network is a useful tool to explore molecular interactions as well as to identify potential key biomarkers. Based on the CC-related genes of OC, we constructed a PPI network by the Search Tool for the Retrieval of Interacting Genes (STRING) database with a combined score of higher than 0.7 (high confidence) (Zhang et al., 2021). The Cytoscape software (version 3.8.2, http://www.cytoscape.org) was then utilized for the visualization. Next, we performed sub-module analysis with the MCODE plugin. Potential key genes of CC were defined as those with a clustering coefficient of 1 in the entire PPI network.

### 2.5 Function enrichment

The DAVID database was used to conduct the function enrichment for the CC-related genes and the key genes in the most significant gene module. The Gene ontology (GO) database, which is comprised of MF, BP, and CC, is used for performing gene annotation, and the Rectome database was used to analyze the major pathways. The “ggplot2” package was used for data visualization.

### 2.6 Assessment of prognostic and clinical significance

For survival evaluation, we depicted the Kaplan-Meire plots with log-rank tests for OS or DSS by the optimized expression value, which was carried out on the 378 OC patients (Guo et al., 2021; Tang et al., 2022b), who were subsequently classified into different risk groups. To unveil the correlations between the expression of these key genes and clinicopathological traits, we compared their expression levels between different subgroups divided by clinicopathological features including age, stage, grade, tumor status, and lymphatic invasion status. Pearson correlation matrices were further computed to evaluate the co-expression of these crucial genes of CC. Besides, we use boxplots and ROC curves to assess the predictive capabilities of these key genes for discriminating the distinct CCI groups. The online cBioPortal database (http://www.cbioportal.org/) was used to verify their genetic alterations with an oncoprint diagram.

### 2.7 Immune infiltration profile

To explore the relationships between the potential key genes and TIME, we utilized the CIBERSORT algorithm (Newman et al., 2015; Tang et al., 2022a) to estimate the relative abundance of 22 immune cell types in each OC sample based on the TCGA-OV dataset. The relative abundance of immune cells in different CCI groups was computed and presented by a heatmap plot. Spearman correlation analysis was used to assess the relevance of the key genes’ expression and immune cell infiltration.

### 2.8 Prediction of upstream regulations-transcript factor and miRNA

MiRNet 2.0, an up-to-date online platform that illustrates “multiple-to-multiple” relationships and functional interpretation was used to examine the upstream transcription factors of key genes. We selected the JASPAR database to screen the possible transcription factor-key gene interactions in the present study. Moreover, the upstream miRNAs of the key genes were putatively predicted with miRTarBase 8.0, which is a powerful tool to predict miRNA-target interactions (MTIs) that were verified by multiple cell-based experiments. The transcription factor-key gene interactions and miRNA-key genes interactions were visualized by the Cytoscape software.

## 3 Results

### 3.1 Survival and differential analysis of 12 CCGs in OC

To achieve a systematic understanding of the circadian clocks in OC, we first selected 12 well-known circadian genes in this study, namely, *ARNTL*, *CLOCK*, *PER1*, *PER2*, *PER3*, *CRY1*, *CRY2*, *NR1D1*, *NR1D2*, *RORA*, *RORB*, and *RORC*. Based on the GEPIA online database, we preliminarily compared the mRNA levels of these genes in OC tissues and adjacent ovary tissues (Supplementary Figure S1A). As a result, the mRNA level of *RORC* was upregulated, while the expression of *ARNTL*, *CRY2*, *NR1D1*, *PER1*, *PER3*, and *RORA* was decreased in OC tissues versus normal tissues. This evidence demonstrates that core circadian clock genes are widely altered at the mRNA level in OC. To analyze the prognostic value of circadian clock genes in OC, we performed Kaplan-Meier analysis and log-rank tests of the 12 CCGs for OS of OC. As shown in Supplementary Figure S1B, we found that *ARNTL* (*p* = 0.021), *CRY1* (*p* = 0.0074), *CRY2* (*p* = 0.045), *NR1D1* (*p* = 0.0035), *NR1D2* (*p* = 0.0062), *PER1* (*p* = 0.014) and *PER2* (*p* = 0.025) were significantly associated with the OS of OC. These results suggest a possible link between CC and the progression and prognosis in OC.

### 3.2 CCI was significantly associated with the prognosis and immune status in OC

**FIGURE 2:**
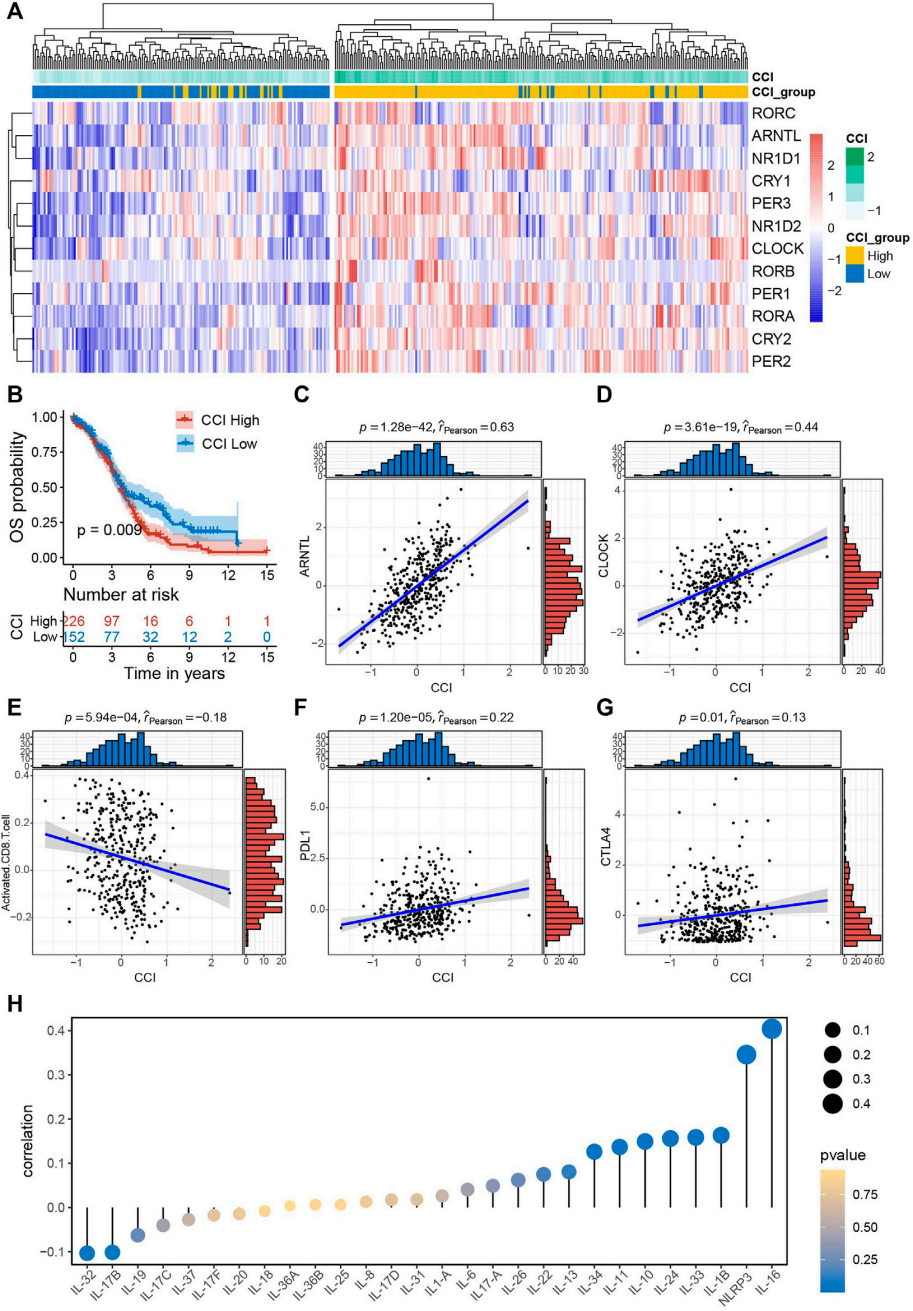
Survival significance of circadian clock index (CCI) and the correlations between CCI and common immune signatures. **(A)** Clustering heatmap of the expression pattern of CCI genes with CCI group annotation in OC. **(B)** Kaplan-Meire plots for OS in the high- and low-CCI group of OC patients. **(C,D)** The strong correlations between CCI and two core CCGs in OC. **(E–G)** The significant correlations between CCI and important immune signatures. **(H)** correlations between CCI and interleukin families.

Firstly, we computed CCIs for all of the OC patients and performed heatmap visualization to show the expression pattern of CCI genes with CCI group annotation in OC (Figure 2A). To evaluate the prognostic power of CCI, we carried out Kaplan-Meier analysis for OS. As a result, OC patients with high expression CCI (*p* = 0.009) had much poorer OS rates than the low CCI group (Figure 2B). Subsequently, we confirmed the correlations between CCI and two core CCGs (*ARNTL*, *CLOCK*) by scatter plots, and found strong positive correlations for both *ARNTL* (*p* = 1.28e-42, rpearson = 0.63) and *CLOCK* (*p* = 3.61e-19, rpearson = 0.44) were linked to CCI in OC (Figures 2C, D). Numerous studies have elaborated on the close correlation between the disruption of CC and the activity of tumor immune status as well as therapeutic effects in various cancers (Yang et al., 2017; Sulli et al., 2019; Kinouchi and Sassone-Corsi, 2020; Xuan et al., 2021; He et al., 2022). To explore the correlations of CCI and immune signatures, we clarified the correlation between CCI and CD8^+^ T cells in OC and found that the CCI was negatively associated with the abundance of CD8^+^ T cells (*p* = 5.94e-04, rpearson = −0.18). Considering the well-known indicative powers of *PDL1* and *CTLA4* for immunotherapeutic response in cancers and their prognostic values in OC (Huang et al., 2017; Huang and Odunsi, 2017), we also examined the correlations between CCI and the expression of them. Interestingly, CCI was positively correlated with the expression of *PDL1* (*p* = 1.20e-05, rpearson = 0.22) and *CTLA4* (*p* = 0.01, rpearson = 0.13) (Figures Figure2E–G). Given the particular relevance and pleiotropic role of interleukins in the progression of cancer, which has attracted great interest of interleukins in translational cancer research recently (Briukhovetska et al., 2021), we further examined the correlations between CCI and several interleukin families. As a result, *IL-16* was with the strongest correlation with CCI, and significant associations were also observed for *NLRP3* and *IL-1* superfamily (including *IL-1β* and *IL-33*) (Figure 2H; Supplementary Figure S1). Besides, we also found significant correlations between CCI and several steroid hormones-related genes (*SRD5A2*, *HSD17B12*, and *NR3C1*) (Supplementary Figure S2). Collectively, these data demonstrated CCI may provide indicative value for future immunotherapy in OC.

### 3.3 Identification of key biomarkers and CC-related genes via WGCNA

**FIGURE 3:**
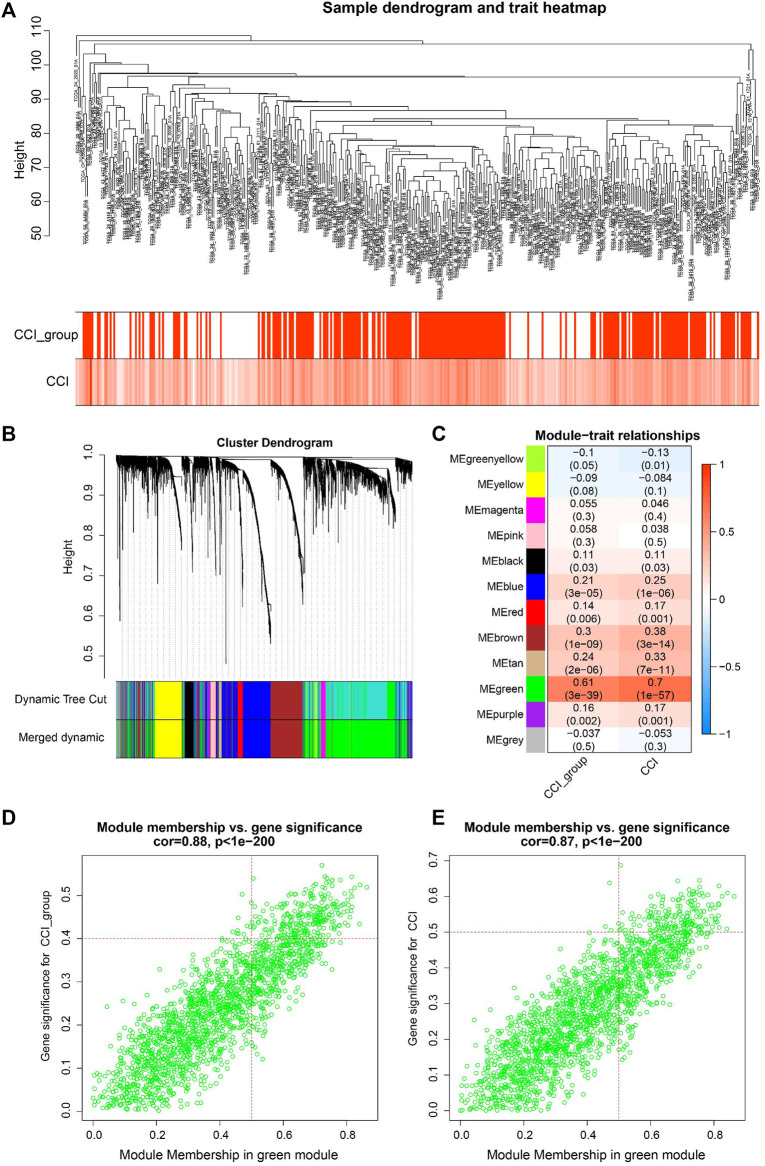
WGCNA for identification of key module and circadian clock-related genes in OC. **(A)** Sample clustering tree with CCI and CCI group annotations. **(B)** Gene clustering dendrogram with the topological overlap matrix (TOM) based dissimilarity. **(C)** Pearson correlation analysis between module eigengenes and CCI and CCI group. **(D,E)** Scatter plots visualizing the gene significance (GS) vs. module membership (MM) of each gene for CCI and the CCI group in the green gene module. CC-related genes were regarded as those with MM > 0.5 and GS > 0.4 for CCI group and GS > 0.5 for CCI in the green module.

WGCNA is a useful approach to construct a co-expression network of genes and to identify significant gene modules or key biomarkers from multiple samples in cancer. In this study, we conducted WGCNA to disclose the most important module associated with CCI and the CCI group. We chose the optimal soft-thresholding power of 4 (scale-free *R* ^2^ > 0.90) as the soft-thresholding to construct a scale-free network (Supplementary Figure S3), followed by the hierarchical clustering of samples using the average linkage method (Figure 3A). Next, the adjacency matrix was produced and transformed into a TOM, which was used to calculate the dissTOM (1-TOM) for evaluating the distance of genes. The dissTOM was subsequently used to conduct hierarchical clustering and to generate dynamic gene modules. After merging highly similar modules by the cut line of 0.3, a total of twelve modules were identified (Figure 3B). The Pearson correlation heatmap showed the green module has the most significant correlation with both the CCI group and CCI (*R* ^2^ = 0.61 and p = 3e−39 for the CCI group, *R* ^2^ = 0.7 and *p* = 13e−57 for CCI, respectively). Thus the green gene module was selected for further study (Figure 3C). Besides, the GS and MM of each gene for CCI and the CCI group in the green module were presented in Figures 3D, E and 178CC-related genes in the green module were picked out with the MM > 0.5 and GS > 0.4 for the CCI group and GS > 0.5 for CCI.

### 3.4 PPI network of CC-related genes

**FIGURE 4:**
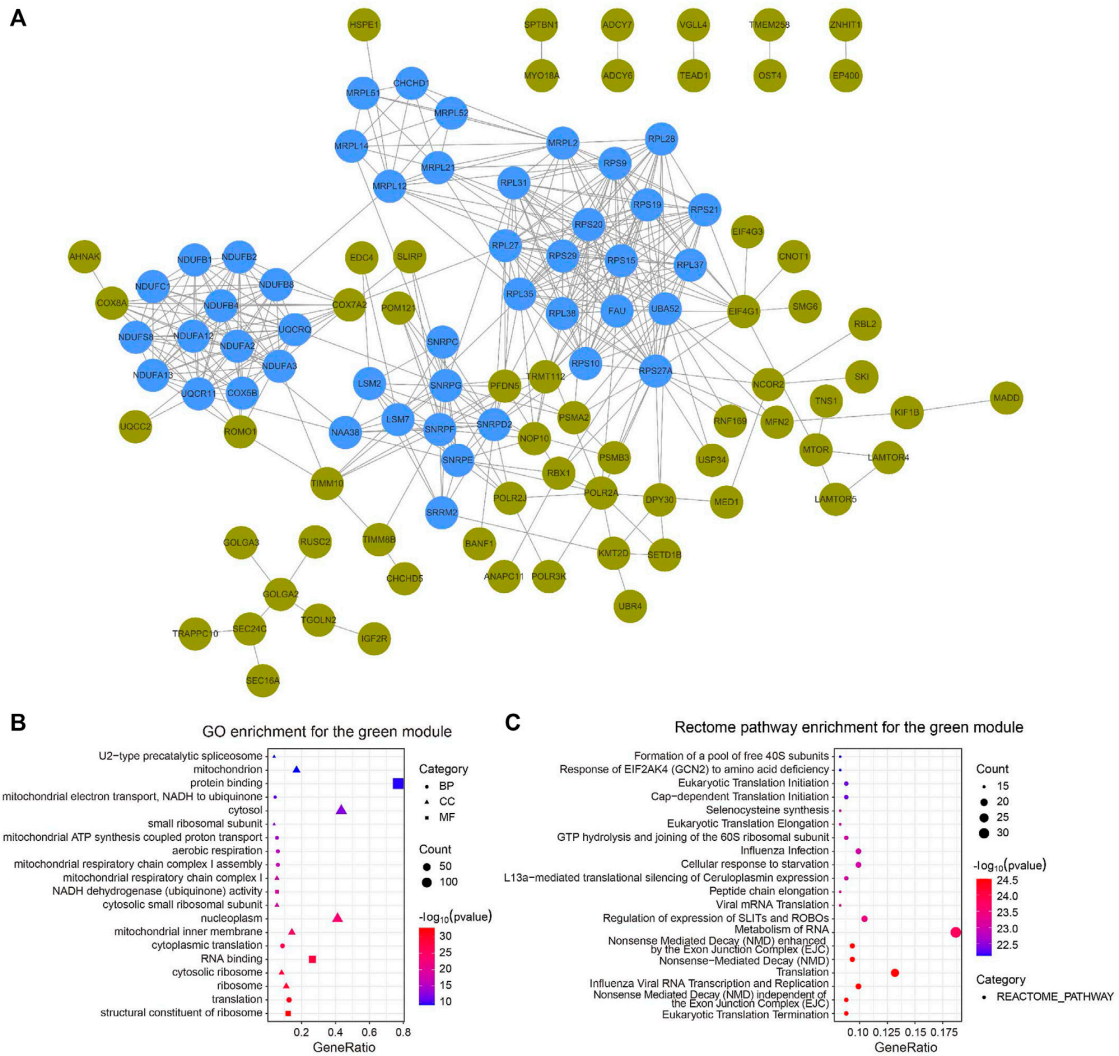
PPI network of circadian clock-related genes. **(A)** PPI network of 107 interactive gene nodes by the STRING database and Cytoscape software. Four clusters (blue nodes) were identified by the MCODE app. **(B,C)** GO **(B)** and Rectome pathway **(C)** enrichment analysis of the 178 circadian clock-related genes. BP, biological process; CC, cellular component; MF, molecular function.

We constructed a PPI network with the CC-related genes in the green module using the STRING online database and the Cytoscape software (Tang et al., 2020), which contained 107 interactive nodes and 424 edges (Figure 4A). The clustering coefficient was 0.554 and the average number of neighbors was 9.258. Four clusters (blue nodes) were identified by the MCODE app with a high network score (>6). To explore the involved biological functions and pathways of the 178 circadian clock-related genes, we conducted GO and Rectome pathway analysis. GO analysis demonstrated these CC-related genes were mostly enriched in the GO terms of protein binding, cytosol, nucleoplasm, RNA binding, etc. (Figure 4B). Meanwhile, pathway analysis suggested the green gene module was mainly related to the Rectome pathway of Metabolism of RNA and so on (Figure 4C).

### 3.5 Identification and function enrichment of key genes of the circadian clock

**FIGURE 5:**
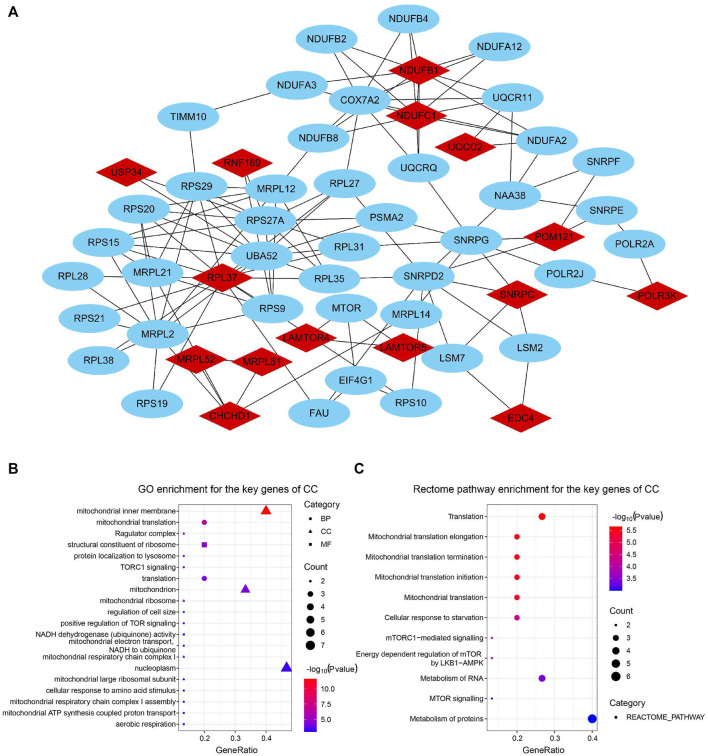
Identification and function enrichment of key genes of the circadian clock. **(A)** 15 key genes of CC with connected genes in the PPI network. **(B,C)** GO **(B)** and Rectome pathway **(C)** enrichment analysis of 15 key genes of CC (Clustering coefficient = 1).

For the identification of the key genes, we selected the top 15 nodes in the PPI network with a clustering coefficient of 1, i.e., *RNF169*, *EDC4*, *CHCHD1*, *MRPL51*, *UQCC2*, *USP34*, *POM121*, *RPL37*, *SNRPC*, *LAMTOR5*, *MRPL52*, *LAMTOR4*, *NDUFB1*, *NDUFC1*, and *POLR3K*. Figure 5A showed the 15 key genes (red) of CC with connected neighbors (blue) in the PPI network. We also employed GO enrichment to elucidate their biological functions and found them remarkably correlated with the GO term of the mitochondrial inner membrane, mitochondrion, and nucleoplasm (Figure 5B). Meanwhile, Rectome pathway analysis showed that the key genes of CC played roles in Mitochondrial translation initiation, Mitochondrial translation elongation, Mitochondrial translation termination, Cellular response to starvation, and Metabolism of RNA and proteins pathways (Figure 5C). The above results indicate their potential hub roles for tumorigenesis.

### 3.6 Aberrant expression and prognostic values of the key genes

**FIGURE 6:**
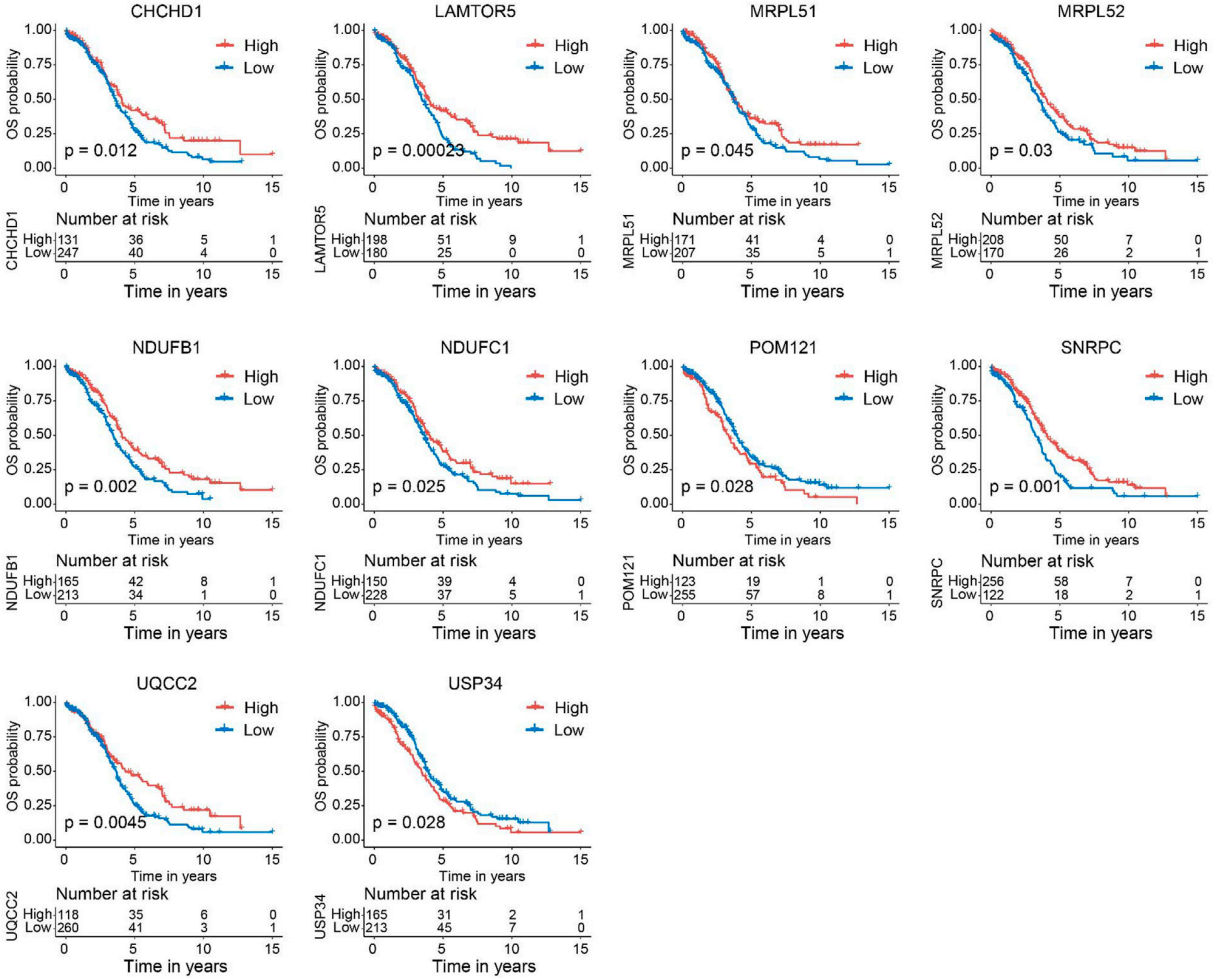
Kaplan–Meier survival curves of the 15 key genes characterizing OS difference with log-rank tests in OC (only significant genes were shown). OS, overall survival.

**FIGURE 7:**
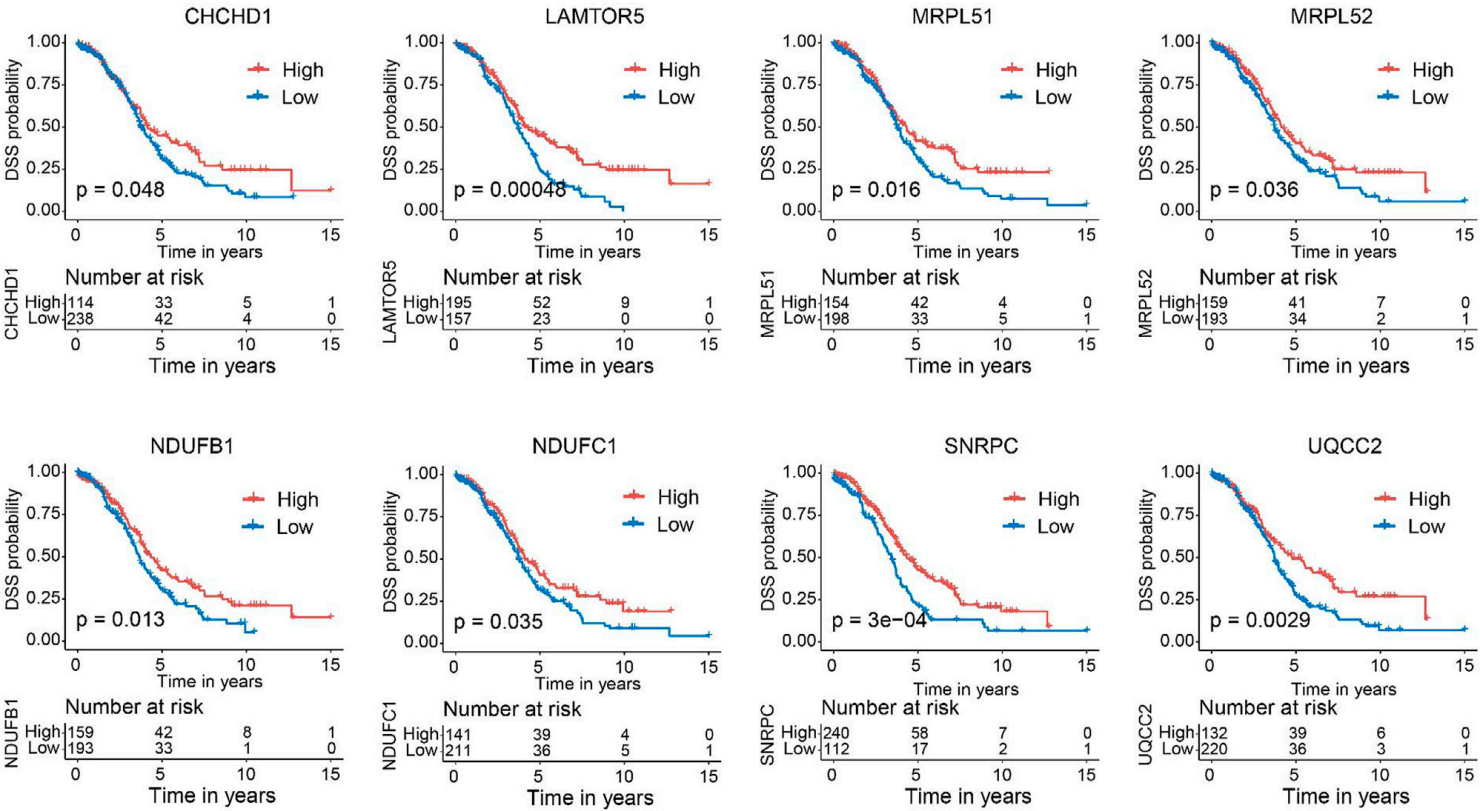
Kaplan–Meier survival curves of the 15 key genes characterizing DSS difference with log-rank tests in OC (only significant genes were shown). DSS, disease-specific survival.

Next, we verified the expression of the filtered key genes in the GEPIA database. *EDC4*, *SNRPC*, and *UQCC2* were found significantly differentially expressed in OC vs. normal tissue (*p* < 0.05). All of them were elevated in OC tissues compared with the normal tissues (Supplementary Figure S4). To evaluate the prognostic powers of these key genes, we examined the 15 key genes in perspective of OS and DSS using Kaplan-Meier analysis and log-rank tests. Among these key genes, the expression levels of *CHCHD1*, *LAMTOR5*, *MRPL51*, *MRPL52*, *NDUFB1*, *NDUFC1*, *POM121*, *SNRPC*, *UQCC2*, and *USP34* were significantly linked to OS of OC (Figure 6). For DSS survival analysis, We found that low expression of *CHCHD1* (*p* = 0.048), *LAMTOR5* (*p* = 0.00048), *MRPL51* (*p* = 0.016), *MRPL52* (*p* = 0.036), *NDUFB1* (*p* = 0.013), *NDUFC1* (*p* = 0.035), *SNRPC* (p = 3e-04), *UQCC2* (*p* = 0.0029) was significantly associated with worse prognosis (Figure 7). These data showed the good potential of the 15 key genes for the development of prognostic indicators in OC.

### 3.7 Correlation analysis of the key genes

**FIGURE 8:**
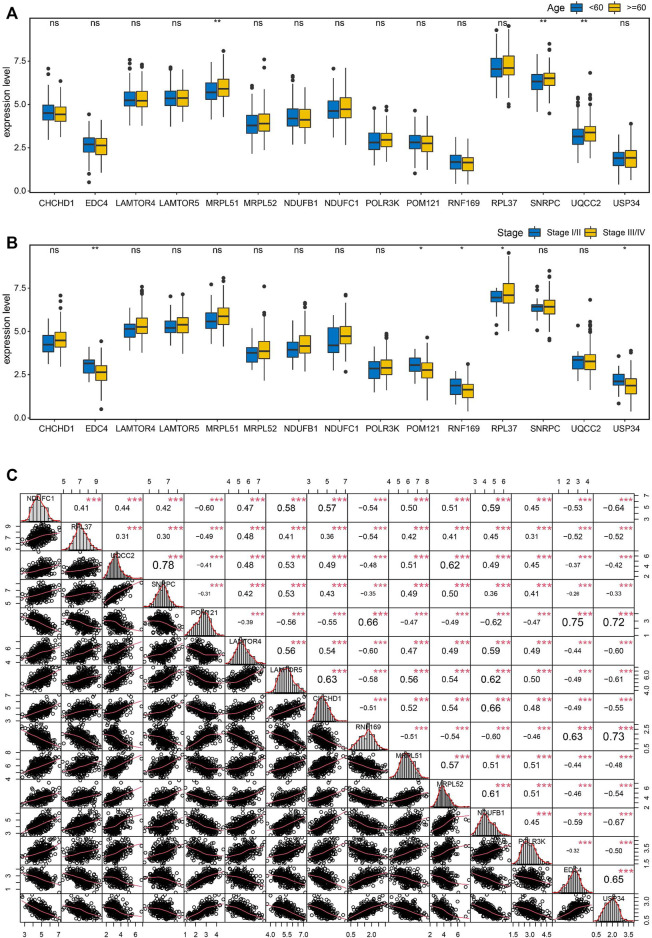
Correlation analysis of the key genes in OC. **(A)** Boxplots showing the correlations of the key genes with age **(A)** and stage **(B)**. **(C)** Pearson correlation matrices of expression values of the key genes. *p < 0.05, **p < 0.01, ***p < 0.001, ns, no significance.

To seek the clinical relevance of these key genes, we compared the expression levels of all 15 key genes between subgroups by clinical variables including age, stage, grade, tumor status, and lymphatic invasion status, but all showed no significant differences for most of these features except age and tumor stage (Figures 8A, B). Moreover, Pearson correlation analysis coupled with statistical significance demonstrated strong correlations between the expression of these key genes, implying their tight connections (Figure 8C).

### 3.8 All of the key genes were highly indicative of the CCI group in OC

**FIGURE 9:**
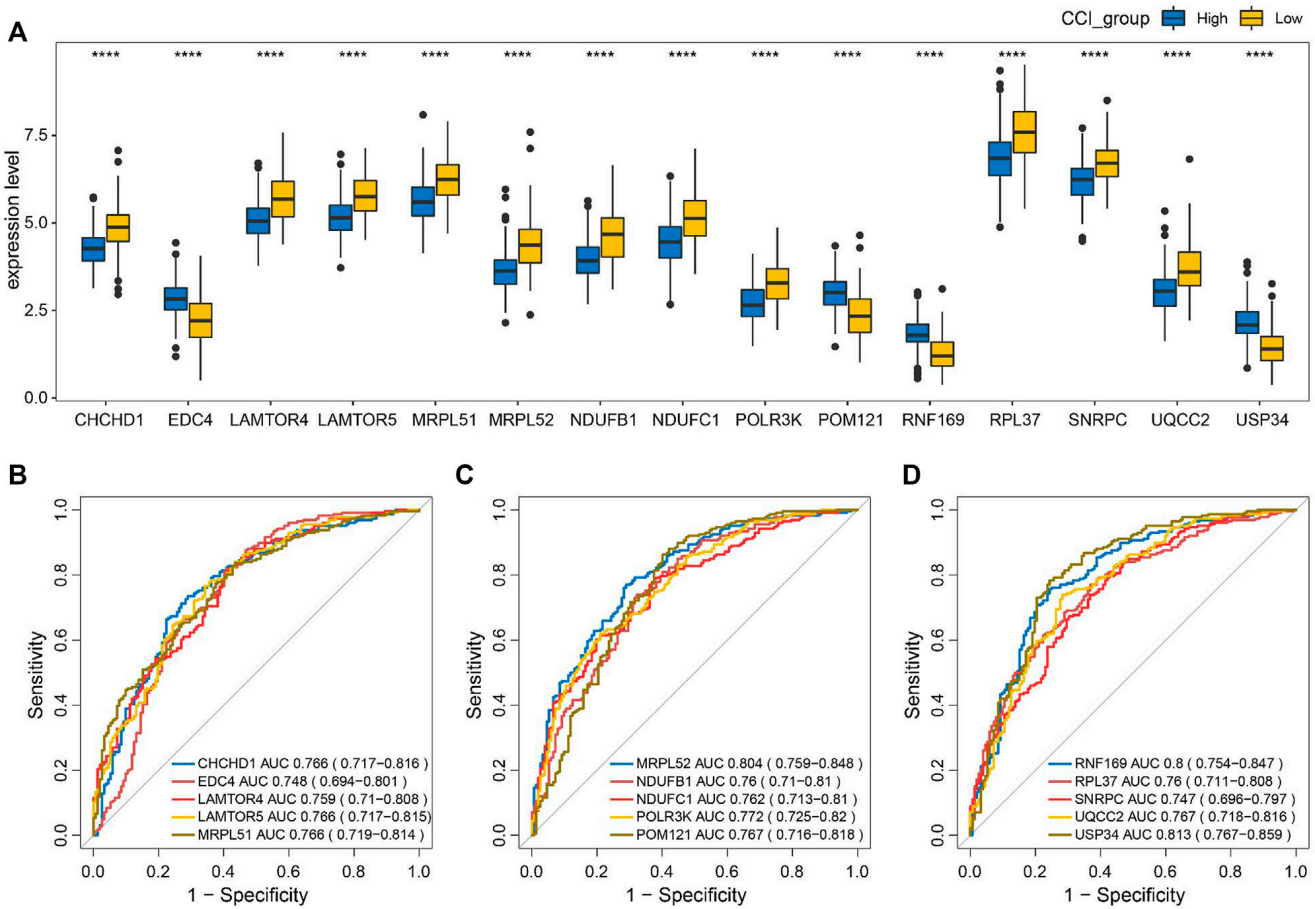
Discriminating capacities of the key genes between different CCI groups. **(A)** Comparison of expression levels of the 15 key genes in different CCI groups. **(B–D)** ROC curves for the evaluation of the discriminating capacities for the 15 key genes between different CCI groups.

To evaluate the discriminating capacities of the 15 key genes in different CCI groups, we compared the transcription expression levels of the 15 key genes between the high-CCI group and the low-CCI group. Consequently, all of the 15 key genes showed statistical differences between the high CCI group and the low CCI group (Figure 9A). The above findings prompted us to speculate that these key genes may have good discriminating capacities for different CCI groups, which was verified by plotting ROC curves. The area under the curves (AUCs) of individual key genes exceeds 0.7 (Figures 9B–D). The above findings proved their great potential for discriminating between different CCI groups.

### 3.9 The key genes were significantly correlated to the immune infiltration of OC

**FIGURE 10:**
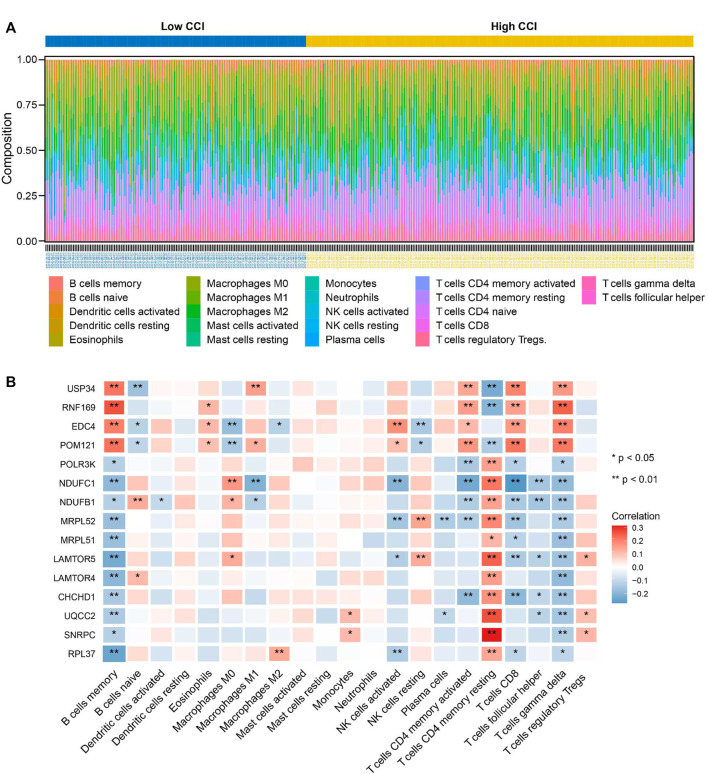
Relationship between key genes of CC and the abundance of immune infiltration cells. **(A)** The landscape of tumor immune infiltration regarding the CCI group in OC. **(B)** Heatmap showing the correlation matrix of the 15 key genes and relative abundance of 22 immune cell types. Red denotes the positive correlation, while blue denotes the negative correlation.

To further explore the correlations between the CCI group and immune infiltration, the relative proportions of 22 immune cell types in OC were calculated by the CIBERSORT algorithm (Figure 10A). Then, we examined the correlation between immune cell infiltration and the expression of the 15 key genes, which indicated that all 15 key genes of the CCI group were significantly associated with most of the immune cells (Figure 10B). At least 8 of them were negatively associated with the infiltration of CD8 cells. And interestingly, we found 11 kinds of immune cells were positively correlated to T cells CD4 memory resting, while they were all negatively correlated to B cells memory in OC. The above results indicated that these key genes might play an important role in the immune status of OC.

### 3.10 Other downstream validations of the key genes

Subsequently, we focused on the potential upstream regulations, including transcription factor and regulative miRNA of the 15 key genes of CC. We predicted the upstream transcription factors of the 15 key genes by the JASPAR database that was integrated into the web-based application of miRNet. A transcription factor-key gene network was constructed and visualized by the Cytoscape software, which includes all 15 key genes and 44 putative upstream TFs (Supplementary Figure S5). Obviously, well-known oncogenic transcription factors such as *JUN*, and *E2F1* were involved in the expression regulation of the key genes. Considering the critical role of miRNA in regulating gene expression and in tumorigenesis (Zhang et al., 2016), the upstream miRNAs of the 15 key genes of CC were predicted by the miRTarBase 8.0 database. The miRNA-target network was next constructed and visualized by the Cytoscape software, which includes 13 of the 15 genes and 242 putative upstream miRNAs (Supplementary Figure S6). Hence, these data could lay a firm basis for the role of the CC-related key genes in the further exploration of the molecular mechanisms of OC.

To obtain a deeper underlying of the 15 key genes from the DNA layer, we analyzed the gene mutation landscape of the 15 key genes of CCI with the aid of cBioPortal. Supplementary Figure S7 showed all kinds of common genetic mutations of the 15 key genes including inframe-mutation, Missense-mutation, truncating-mutation, amplification, deep deletion, mRNA high, and mRNA low. Among these genes, *RNF169* was the most mutated one, which exhibited a genetic mutation rate of 14% in the TCGA-OV cohort, other frequently mutated key genes including *EDC4* (11%), *CHCHD1* (10%), *MRPL51* (10%), *UQCC2* (10%), and *USP34*(10%), respectively. These results may disclose the molecular mechanisms of the dysregulation of these key genes from the genomic level.

## 4 Discussions

Ovarian cancer is the most common malignancy among all gynecological cancers, and its histopathological classification mainly includes epithelial ovarian cancer, sex cord-stromal tumors, and germ cell tumors, among which epithelial tumors are the most common, accounting for more than 90%. Ovarian cancer is a malignant tumor of the female reproductive system with the highest mortality rate, and the current treatment is mainly surgical treatment supplemented by radiotherapy, chemotherapy, immunotherapy, etc., but the overall survival rate of ovarian cancer is still poor. Biorhythms play an important role in regulating numerous physiological activities of mammals, including cell growth, secretion, and metabolism (Reppert and Weaver, 2002). Numerous studies have confirmed a close link between biorhythm and cell cycle and apoptosis (Fu et al., 2002). Abnormal expression of cell cycle-related genes will cause cell cycle disorder, resulting in an imbalance of cell growth and apoptosis, which can lead to the occurrence of cancer (Viallard et al., 2001). The ovaries have specific cyclical activities, which are closely related to human reproductive health, but the biorhythm and the occurrence and regulation mechanism of ovarian cancer are not clear.

The change in the gene dose of the circadian clock and loss of control of gene doses in the linked transcription-translation feedback loop lead to disruption of the circadian clock, and thus develop into tumors (Lee et al., 2011). The ovary is one of the important reproductive organs of mammals, and the rhythmic expression of circadian clock genes is found in the intact ovary (Fahrenkrug et al., 2006). In infertility studies, it has been found that the reproductive cycle of the ovaries is affected by circadian rhythms, and their rhythms are coordinated and synchronized by neurological and endocrine tissues (Gallego et al., 2006; Etchegaray et al., 2009). This coordination is facilitated by gene expression and cellular physiology at all levels of the hypothalamic-pituitary-ovary (HPO) axis. The expression of circadian clock genes has been observed in the endocrine regulatory axis of the ovaries, and the circadian clocks at all levels coordinated and synchronized with each other to maintain normal reproductive behavior (Alvarez et al., 2008). In peripheral ovarian tissues, changes in the timing of circadian clock gene expression may be the result of hormonal imbalances associated with polycystic ovary syndrome (PCOS) (Amaral et al., 2014). The reproductive function of the ovaries is richly related to biorhythms, so the relationship between ovarian cancer and biorhythms is of great interest to us.

We comprehensively identified 15 key genes by elucidating the relationship between circadian clock control genes and survival, tumor stage, and subtype in ovarian cancer patients using various statistical and bioinformatics methods. These genes play a role in translation, mitochondrial translation initiation, extension, termination, and protein metabolism pathways that influence tumor progression and development. For example, *POM121* forms the core component of the nuclear pore complex, mediating the transport of molecules in and out of the nucleus, and blocking the function of *POM121* can inhibit the nuclear localization of transcription factors including *MYC* and *E2F1*, thereby reducing the growth rate of prostate cancer tumors (Rodriguez-Bravo et al., 2018). The expression levels of mRNA and protein of *POM121* in colon cancer, oral squamous cell carcinoma (Ma et al., 2019), lung cancer (Zhang et al., 2020), and laryngeal cancer tissues are also found to be higher than in adjacent tissues (Zhao et al., 2020). *EDC4* is a well-known regulator of mRNA decapping, which was related to mRNA decapping, genome stability, and sensitivity of drugs. *EDC4* plays a key role in homologous recombination by stimulating end resection at double-strand breaks. Lack-of-function mutations in *EDC4* were detected in breast cancer (Gudkova et al., 2012). *SNRPC* is one of the specific protein components encoding U1 small ribonucleoprotein (snRNP) granules and is upregulated and prognostically related in liver cancer patients (Hernandez et al., 2018). The same trend of changes in the *POM121*, *EDC4*, and *SNRPC* genes was also found in ovarian cancer.

The linkage between mitochondrial alteration and cancer has been uncovered in multiple cancer types including liver (Huang et al., 2016), lung (Qi et al., 2020), colon (Tailor et al., 2014), breast (Zhao et al., 2013), and ovarian (Kingnate et al., 2018) cancers. In this study, several key genes of CC such as *CHCHD1*, *MRPL52*, *MRPL51*, *UQCC2*, *NDUFB1*, and *NDUFC1* are all important regulators in the mitochondrial respiratory chain, which had significant effects on the expression of mitochondrially encoded proteins. For example, MRPs play an important role in the synthesis of the basic subunits of the oxidative phosphorylation (OXPHOS) complex, *MRPL51*, and *MRPL52* may interact with Mhr1 to regulate mtDNA repair (Cai et al., 2021). The protein encoded by *UQCC2* affects insulin secretion and mitochondrial ATP production by regulating mitochondrial respiratory chain activity. *NDUFB1* and *NDUFC1* are auxiliary subunits of NADH dehydrogenase (complex I), responsible for transporting electrons from NADH to the respiratory chain necessary for oxidative phosphorylation. The downregulation of *NDUFC1* expression significantly inhibits the proliferation of hepatoma cells (Sahu et al., 2019) and increases the number of apoptotic cells in liver cancer (Han et al., 2022). We suspect that circadian clock genes may regulate the function of these genes through a transcription-translation feedback loop, thus having a potentially pivotal role in tumorigenesis and development.

Given the circadian rhythm regulation of cancer-related physiological systems such as immune response, cell cycle, and apoptosis, immune therapy may become a promising trend in tumor treatment (Dumbell et al., 2016). The immune system plays a vital role in immune surveillance, with immune cells penetrating the tumor microenvironment and helping to regulate tumor progression. Immune cells are the cellular basis of immunotherapy, and a deeper understanding of immune infiltration in the tumor immune microenvironment can reveal the underlying molecular mechanism and provide new strategies for improving the efficacy of immunotherapy (Wu J. et al., 2019). Immunoinvasive studies have shown that the tumor immune microenvironment plays a key role in cancer progression and influences clinical outcomes in cancer patients (Zhang and Zhang, 2020). Studies have shown that the induction of immune response and the regulation of autoimmunity are affected by the regulation time of the immune system (Sutton et al., 2017). Studies have also shown that the body’s immune system fluctuates rhythmically with circadian rhythms (Wang et al., 2023), and the body’s internal clock (biorhythm) has an important impact on the ability of immune cells to recognize cancer cells and promote their clearance, a discovery that may be used to improve the effectiveness of cancer treatment.

The composition of immune cells in the tumor microenvironment also affects cancer prognosis (Ock et al., 2016; Guo et al., 2022). Studies have shown a strong correlation between the circadian clock and immune cells (Zhou et al., 2020), and in many tumors, and downregulation of core clock genes (PER1, PER2, PER3, CRY1, and CRY2) expression is significantly associated with T cell failure and upregulation of immunosuppressive molecules (Wu Y. et al., 2019). Our study found that CCI in OC was negatively correlated with CD8^+^ T cell abundance, which coincided with the change in response intensity of CD8 T cells during vaccination with time (Nobis et al., 2019). At the same time, melanoma studies have found dendritic cells and CD8^+^ T cells to exert circadian rhythm anti-tumor functions and control the volume of melanoma (Qian et al., 2021). The above observations suggest that CCI may be a candidate for future immunotherapy in OC. Interestingly, further analysis found that the selected key genes were closely linked to immune infiltration. Studies have shown that *POM121* inhibits macrophage inflammatory response by reducing NF-κB phosphorylated P65 nucleation, which is associated with tumor lymph node metastasis staging (Zhang et al., 2020), and the expression of *USP34* in diffuse large B-cell lymphoma is significantly higher than that in reactive lymphoid hyperplasia (Li et al., 2018). *USP34* can also promote the proliferation and migration of pancreatic cancer cells by upregulating p-AKT and p-PKC proteins (Gu et al., 2019). Similarly, the *EDC4* and Dcp1a complexes are involved in the post-transcriptional regulation of *IL-6*, thereby affecting the function of immune cells (Seto et al., 2015). In this study, the expression levels of 15 key genes were analyzed concerning the proportion of immune cell types. As a result, 11 key genes were negatively associated with T cell gamma delta, most of which were also negatively correlated with the infiltration of CD8 T cell. The left four genes (*USP34*, *RNF169*, *EDC4*, and *POM121* were positively correlated with the infiltration of CD8 T cell, T cell gamma delta, B cells memory, and T cells CD4 memory activated). These data in turn strengthened the utility of CCI as a potential immunotherapy target. Consequently, the combination of drug treatment timing and circadian rhythm may be a new strategy to improve the therapeutic responses and improve the survival rate of patients with OC. Therefore, the circadian rhythm of cancer immune surveillance is not only critical for controlling tumor size but can also be used to guide scientists and doctors to administer cancer immunotherapy to patients at the right point in time, pointing to a new direction for cancer treatment. The expression of CCI-related genes and the identified key CC genes are expected to be taken into consideration in clinical practice for determining a personalized treatment regimen in patients with OC, and the CCI or key CC genes-based drugs and small compounds might be further designed for targeted therapy.

Upstream transcription factors or miRNAs of the key CC genes were comprehensively predicted, and well-known oncogenic transcription factors were involved. Genomic alterations revealed frequent somatic mutations of *RNF169*, *EDC4*, *CHCHD1*, *MRPL51*, *UQCC2*, and *USP34*, which were poorly studied in OC, thus providing new insights into the molecular regulation of these genes from the genomic layer.

In summary, we comprehensively identified 15 key CC genes with clinical implications, which not only improve the understanding of the critical role of CC in tumor initiation and progression, as well as the tumor immune microenvironment but also provide novel insight for future biomarkers or molecular classification development.

## Data Availability

Publicly available datasets were analyzed in this study. This data can be found here: https://portal.gdc.cancer.gov/.

## Abbreviations

CC, circadian clock; OC, ovarian cancer; CCI, circadian clock index; WGCNA, weighted gene co-expression network analysis; TCGA, The Cancer Genome Atlas; PPI, protein-protein interaction; TME, tumor microenvironment; TIME, tumor immune microenvironment; OS, overall survival; DSS, disease-specific survival; MAD, median absolute deviation; TOM, topological overlap matrix; GS, gene significance; MM, module membership; STRING, the Search Tool for the Retrieval of Interacting Genes; MTIs, miRNA-target interactions; CCGs, CC-related genes; GO, Gene ontology; AUCs, area under the curves.
