# Supplementary material for: Integrative analysis of circadian clock with prognostic and immunological biomarker identification in ovarian cancer
Source: Front Mol Biosci. 2023 Jun 20;10:1208132. doi: 10.3389/fmolb.2023.1208132 (PMC10318361; doi:10.3389/fmolb.2023.1208132)
Supplement: Supplementary file 1 [file DataSheet1.docx]

Supplementary Material

**Supplementary Table 1 Correlations between CCI and interleukin families**

| **Interleukins** | **correlation** | **pvalue** |
| --- | --- | --- |
| IL1A | 0.02636322 | 0.609385488 |
| IL1B | 0.163385782 | 0.001435048 |
| IL33 | 0.15863879 | 0.001976846 |
| IL18 | -0.007766812 | 0.880365231 |
| IL37 | -0.027337216 | 0.596226343 |
| IL36A | 0.00397852 | 0.938547662 |
| IL36B | 0.006353709 | 0.902010223 |
| IL6 | 0.040715896 | 0.429929022 |
| IL11 | 0.136854904 | 0.007710313 |
| IL31 | 0.018203611 | 0.724256583 |
| IL10 | 0.149317489 | 0.003616852 |
| IL19 | -0.062619677 | 0.224508822 |
| IL20 | -0.014477248 | 0.779053751 |
| IL22 | 0.075045182 | 0.145318816 |
| IL24 | 0.156066769 | 0.002343087 |
| IL26 | 0.062284198 | 0.22700653 |
| IL17A | 0.049192702 | 0.340172006 |
| IL17B | -0.101366207 | 0.048914935 |
| IL17C | -0.040259845 | 0.435117252 |
| IL17D | 0.017698826 | 0.731604688 |
| IL17F | -0.017355778 | 0.736612473 |
| IL25 | 0.006467642 | 0.900262162 |
| NLRP3 | 0.345883494 | 4.61E-12 |
| IL8 | 0.012956031 | 0.801759739 |
| IL13 | 0.080759415 | 0.116997027 |
| IL16 | 0.404115358 | 2.78E-16 |
| IL32 | -0.103272082 | 0.044796862 |
| IL34 | 0.126263914 | 0.014027443 |


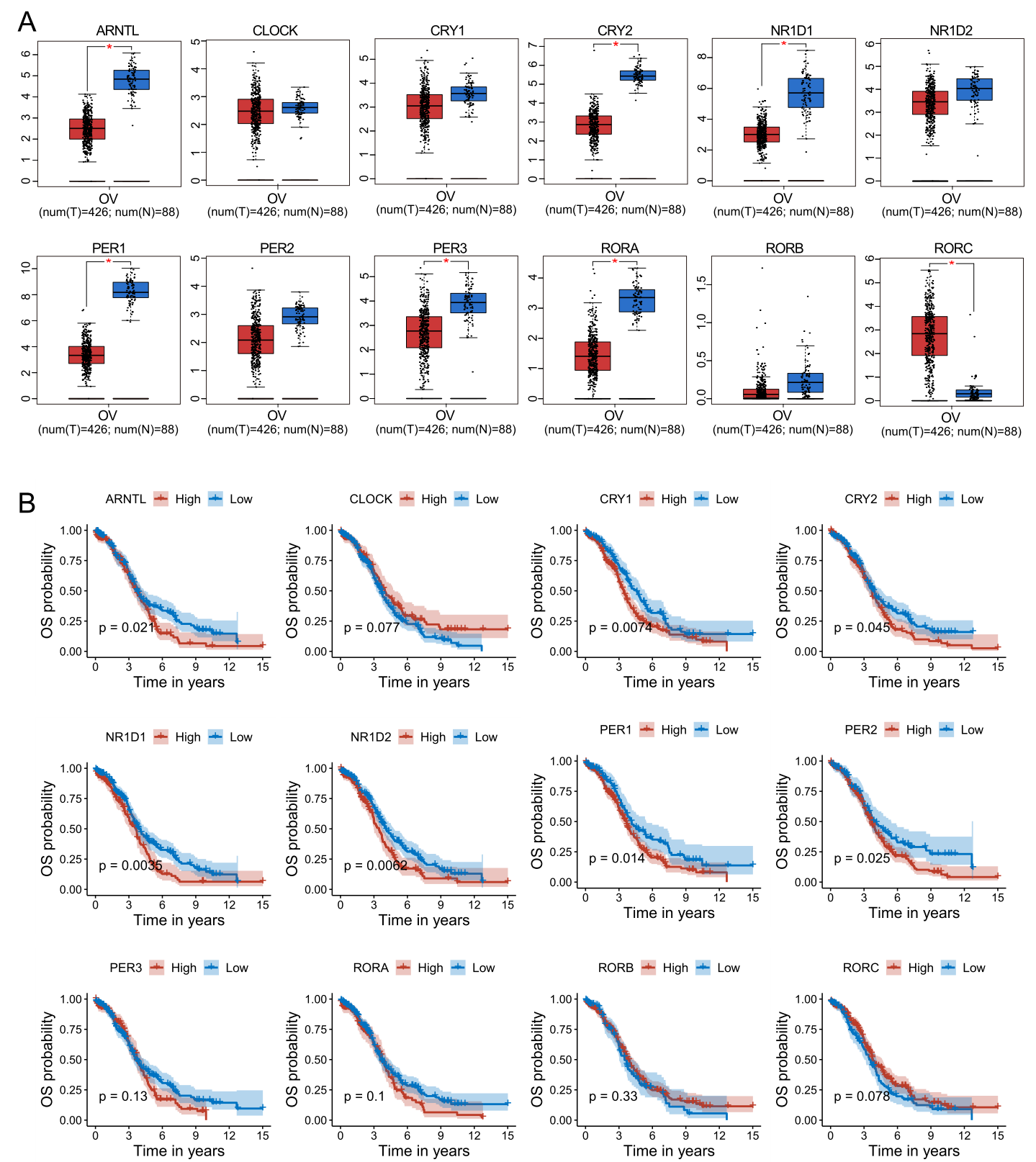


**Supplementary Figure 1 Survival and differential analysis of 12 circadian clock genes (CCGs) based on the TCGA database.** (A) The mRNA expression levels of 12 circadian clock genes in OC of GEPIA. (B) Kaplan–Meier survival plots of 12 circadian clock genes for overall survival (OS) in OC.


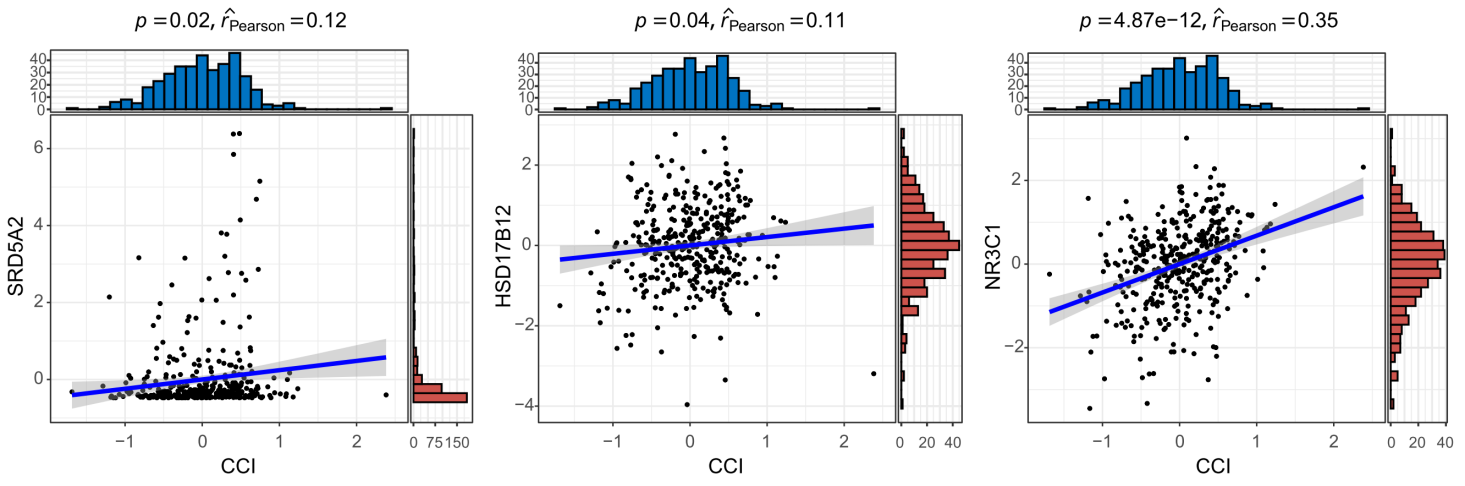


**Supplementary Figure 2 Significant correlations between CCI and steroid hormones-related genes (*SRD5A2*, *HSD17B12*, and *NR3C1*).**

.
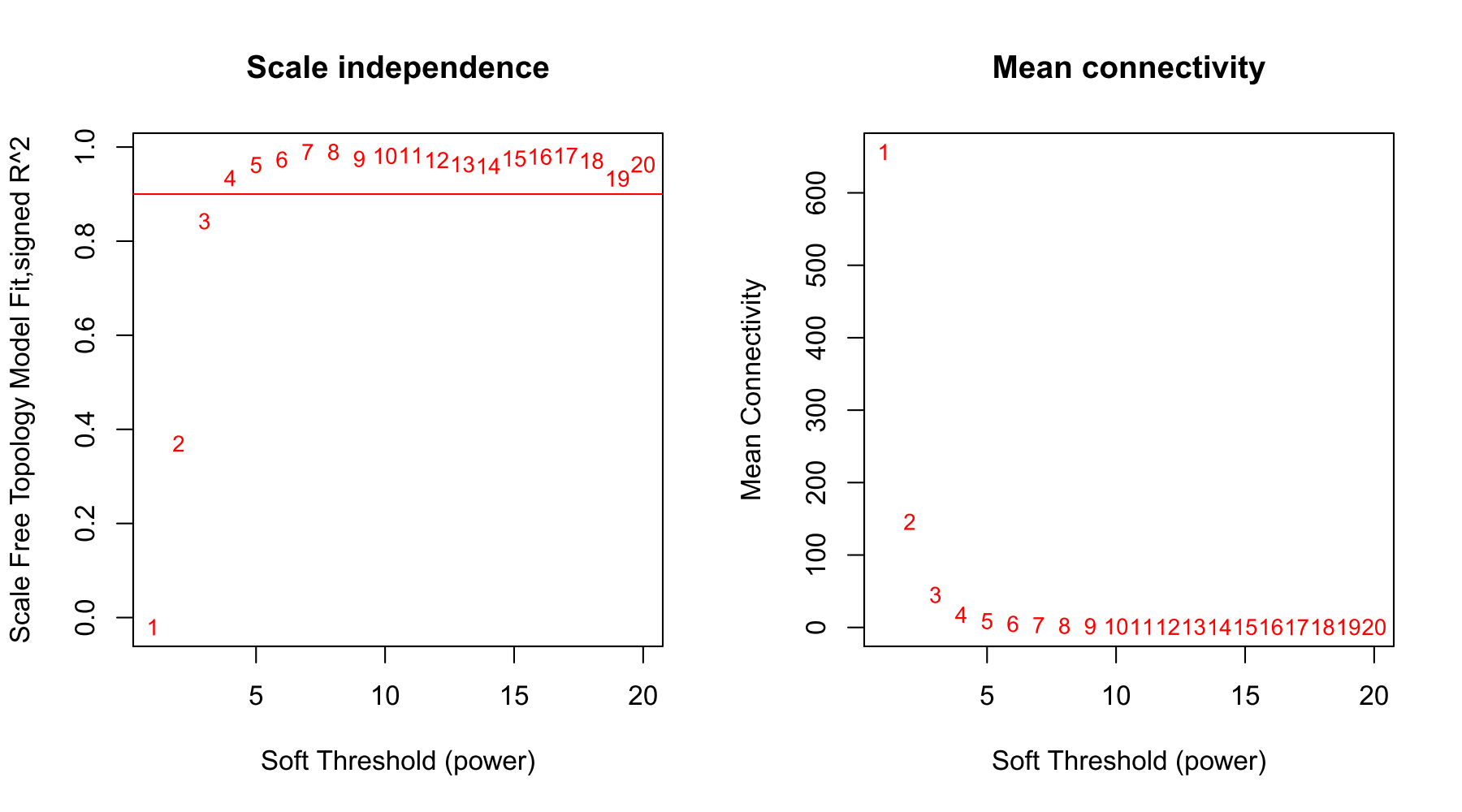


**Supplementary Figure 3 Selection of the optimal soft-thresholding power for the scale-free network.**


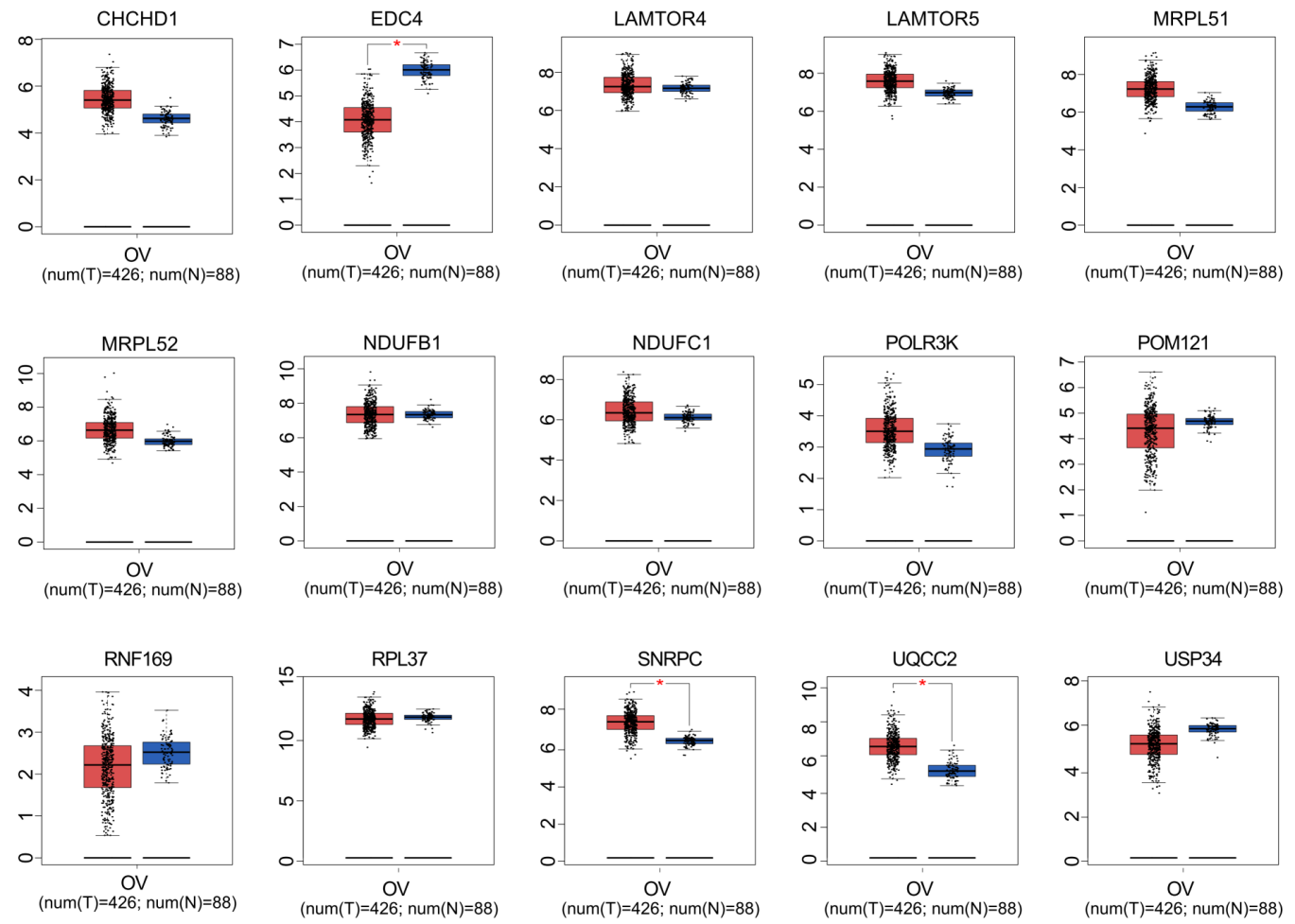


**Supplementary Figure 4 Differential analysis of 15 key genes by GEPIA database.**


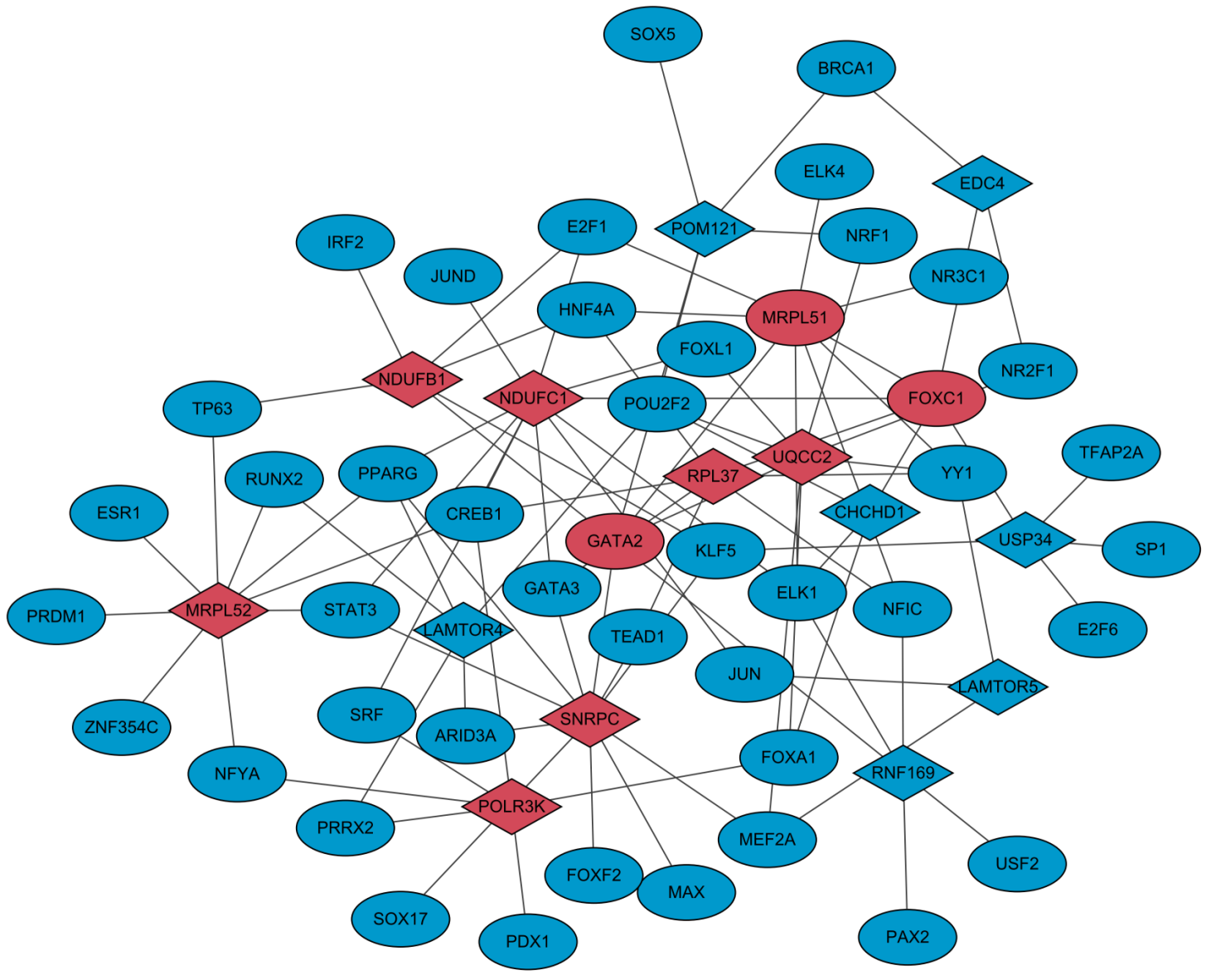


**Supplementary Figure 5 Transcript factor-key gene network prediction. Ellipse nodes represent TF, and diamond nodes represent key genes. Red nodes represents the hub nodes with the highest degree.**


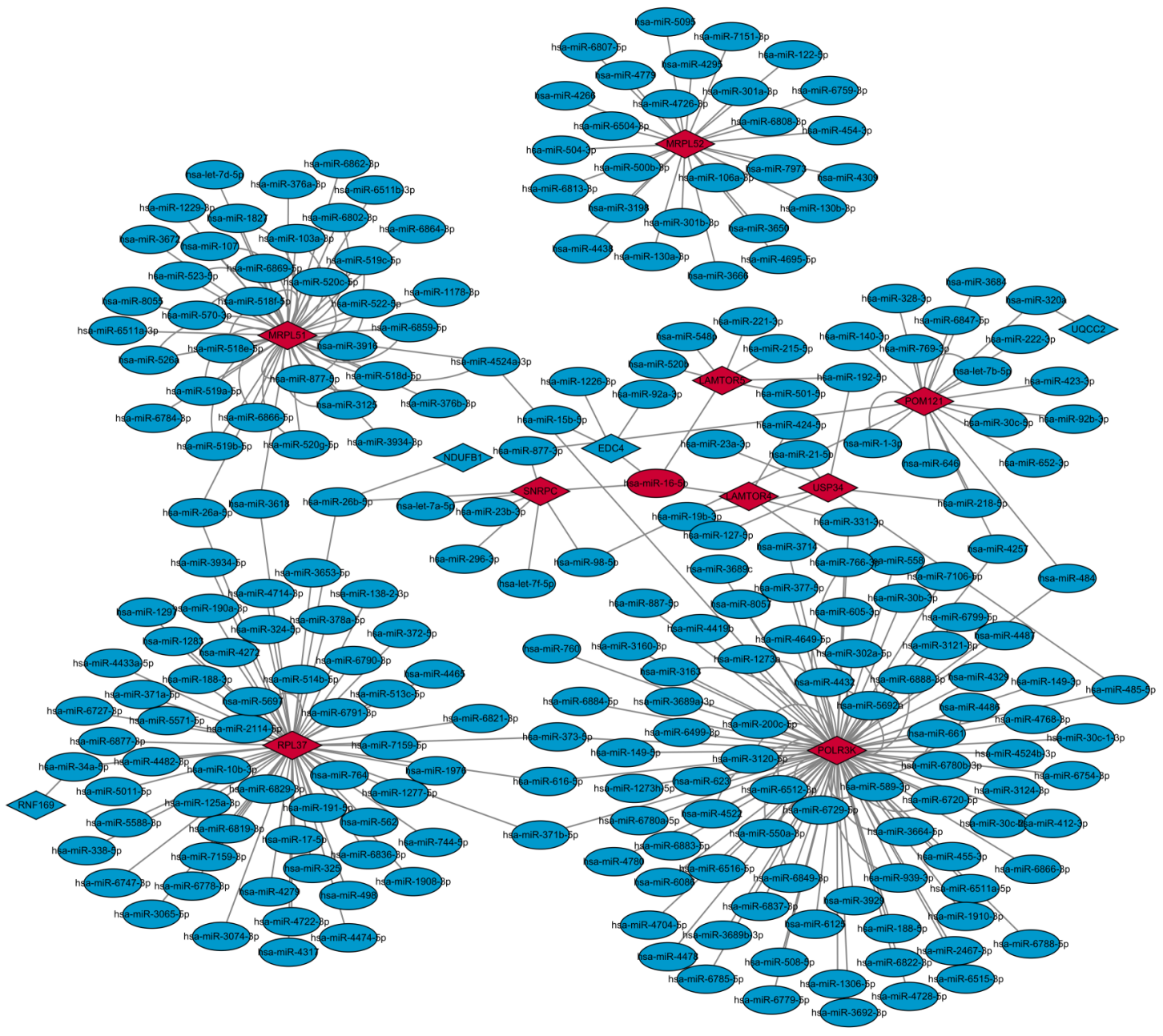


**Supplementary Figure 6 MiRNA-target network prediction. Ellipse nodes represent TF, and diamond nodes represent key genes. Red nodes represents the hub nodes with the highest degree.**


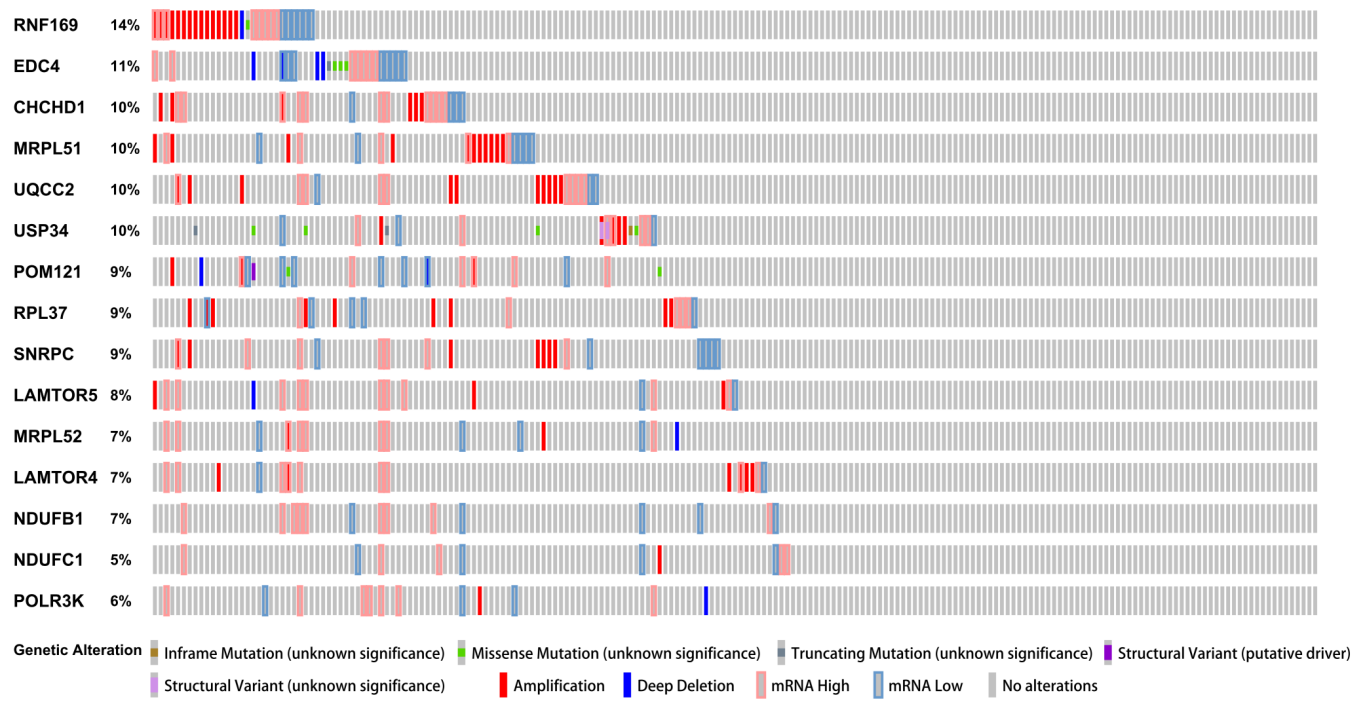


**Supplementary Figure 7 Oncoprint of the 15 key genes of CC.**
